# I-GLAD: a new strategy for fabricating antibacterial surfaces

**DOI:** 10.1186/s11671-024-03959-0

**Published:** 2024-01-25

**Authors:** Chuang Qu, Jesse Rozsa, Mark Running, Shamus McNamara, Kevin Walsh

**Affiliations:** 1 Department of Electrical and Computer Engineering, University of Louisville, Louisville, KY 40292 USA; 2 Department of Biology, University of Louisville, Louisville, KY 40292 USA

**Keywords:** I-GLAD, Glancing angle deposition, Antibacterial, Gram-negative, Gram-positive, Nanofabrication, Physical vapor deposition, AMR, Physical bactericidal

## Abstract

The paper uses inverted glancing angle deposition (I-GLAD) for creating antibacterial surfaces. Antibacterial surfaces are found in nature, such as on insect wings, eyes, and plant leaves. Since the bactericidal mechanism is purely physical for these surfaces, the antimicrobial resistance of bacteria to traditional chemical antibiotics can be overcome. The technical problem is how to mimic, synthesize, and scale up the naturally occurring antibacterial surfaces for practical applications, given the fact that most of those surfaces are composed of three-dimensional hierarchical micro-nano structures. This paper proposes to use I-GLAD as a novel bottom-up nanofabrication technique to scale up bio-inspired nano-structured antibacterial surfaces. Our innovative I-GLAD nanofabrication technique includes traditional GLAD deposition processes alongside the crucial inverting process. Following fabrication, we explore the antibacterial efficacy of I-GLAD surfaces using two types of bacteria: *Escherichia coli* (*E. coli*), a gram-negative bacterium, and *Staphylococcus aureus* (*S. aureus*), a gram-positive bacterium. Scanning electron microscopy (SEM) shows the small tips and flexible *D*/*P* (feature size over period) ratio of I-GLAD nanoneedles, which is required to achieve the desired bactericidal mechanism. Antibacterial properties of the I-GLAD samples are validated by achieving flat growth curves of *E. coli* and *S. aureus*, and direct observation under SEM. The paper bridges the knowledge gaps of seeding techniques for GLAD, and the control/optimization of the I-GLAD process to tune the morphologies of the nano-protrusions. I-GLAD surfaces are effective against both gram-negative and gram-positive bacteria, and they have tremendous potentials in hospital settings and daily surfaces.

## Background

Antimicrobial resistance (AMR) has become a global health and development threat that requires urgent multisectoral action to achieve the Sustainable Development Goals (SDGs) [1]. AMR arises from the overuse of antibiotics, which is an economical way to treat bacterial infections. The treatment of bacterial infections becomes more challenging once the bacteria develop resistance and become so-called “superbugs”, such as Methicillin-resistant *Staphylococcus aureus* (MRSA) [2]. The total number of deaths directly and indirectly related to bacterial infections worldwide was estimated to be over 6 million in 2019 [3]. Bacterial infections can occur in hospitals through means such as catheters, ventilators, and bio-implants. Therefore, there is an urgent need to develop new approaches to prevent bacterial infections while limiting the use of antibiotics.

Antibacterial surfaces can be categorized into two groups based on their mechanisms for expelling/killing microbes: physical mechanisms and chemical mechanisms. Naturally occurring antibacterial surfaces, such as cicada wings and copper sheets, exemplify the two fundamental mechanisms of antimicrobial surfaces. The chemical mechanism relies on antibacterial materials present on the surfaces to eliminate microbes. Surfaces coated with metallic substances like titanium/silver [4] and copper [5], as well as those containing organic compounds such as poly(acrylic acid) [6] and CWR11 peptide [7], have been reported as effective against microbes. However, those surfaces have stability and longevity limitations [8]. The physical method of employing nano-protrusions as a bactericidal approach shows promise in killing bacteria and reducing the reliance on antibiotics. Naturally occurring surfaces in nature, such as insect wings (cicada [9–12] and dragonfly [13]), eyes (moth [14]), and plant leaves (lotus [15]), feature nano-protrusion arrays that exhibit bactericidal properties. However, their limited surface areas make them less ideal for practical antibacterial applications. Meanwhile, the naturally occurring designs with known properties provide us with inspiration for developing novel synthetic manufacturable versions with similar antibacterial properties. The physical mechanism, inspired by cicada wings, involves direct physical puncturing of bacteria. The mechanism relies on the surfaces having features that are significantly smaller than the bacteria in size. When bacteria come into contact with these surfaces, the fine features infiltrate the bacteria and eventually disintegrate them. Additionally, there is air trapped between the fine features and the object that lands on the surface, resulting in a superhydrophobic surface with antifouling properties (self-cleaning) [9]. Since bacteria are at the micro-level, the fine features necessary for puncturing them need to be much smaller, typically less than 1 µm and even sub-100 nm. Furthermore, spaces between the fine features are required.

The main challenge lies in whether and how we can engineer larger surface areas consisting of nano-protrusion arrays for practical and cost-effective applications. One approach to fabricating antibacterial surfaces is utilizing natural templates as molds [16–18]. However, the challenge with this strategy is that the available surface areas of these samples are very limited. Achieving fine control and scalability of advanced nanomanufacturing processes are necessary for three-dimensional (3D) nano-protrusion features found on naturally occurring surfaces, such as nanocones and nanopillars. However, traditional top-down nanofabrication methods, particularly for sub-100 nm, high aspect-ratio 3D features, pose serious challenges. Existing state-of-the-art techniques like electron beam lithography involve a trade-off between feature size and scalability/cost, thus limiting the fabrication of artificial antibacterial surfaces. Bottom-up fabrication processes like nano-additive manufacturing using two-photon polymerization (2PP) face challenges in achieving the required sub-100 nm features.

This paper investigates the development of artificial antibacterial surfaces using glancing angle deposition (GLAD), a cost-effective bottom-up nanofabrication process based on physical vapor deposition (PVD). The concept of GLAD was first proposed in the 1950s, and experimental validations have been conducted by the Brett group since the 1990s [19]. GLAD operates on the principle that separated nanofeatures can be formed through directional vapor depositions when the substrate is extremely tilted and rotated, resulting in a ballistic shadowing effect. GLAD offers the capability to create various features, including nanopillars, nanosprings, nanochevrons, and porous structures within membranes. The growth pattern of the nanofeatures to achieve the desired distribution is determined by nucleation sites, also known as seeds, which can be either natural or pre-determined. Pre-determined seeds can modify the distribution of GLAD features to fabricate periodic nanostructures. Commonly used pre-determined seeds include line seeds [20, 21], sphere seeds [22, 23], and dot seeds [24], which are fabricated using other nanofabrication techniques. For example, the use of self-assembled sphere seeds results in a hexagonal distribution of features, allowing for the recreation of hexagonally packed nanopillar cones [12]. One drawback of pre-determined seeds is the cumbersome process of seed preparation, and the area that can be seeded may be limited if the seeding process is not executed properly. On the other hand, natural seeds eliminate the need for that additional step, allowing for the creation of a large area of nanofeatures on the substrate through a single-step deposition process. Although the distribution of the natural seeds is random, GLAD can still produce separated nanopillar arrays using these seeds. Previous publications on GLAD for antimicrobial surfaces from other research groups have mostly focused on depositing metallic nanoparticles, such as Ti nanoparticles, onto surfaces [25–27].

The challenges facing the use of GLAD for 3D nano-protrusions for antibacterial purposes are threefold: firstly, the growth pattern of natural seeds does not align with the desired features; the features tend to grow upwards and wider when given lateral room when the incident angle is high [28], thus producing needles with broad tips. However, for antibacterial surfaces, sharp nanoneedle-like structures are required, with broad bottoms and ultra-fine tips. Secondly, adhesion and robustness pose additional challenges. Thin films often lack the necessary robustness to withstand external mechanical forces, and their adhesion to the substrate might not be ideal. Finally, some of the artificial antibacterial surfaces are only against gram-negative bacteria. For example, cicada wings and mimicries are reported to be effective against only gram-negative bacteria [9, 29]. It is important to have antibacterial surfaces that are effective against both types of bacteria.

**Fig. 1:**
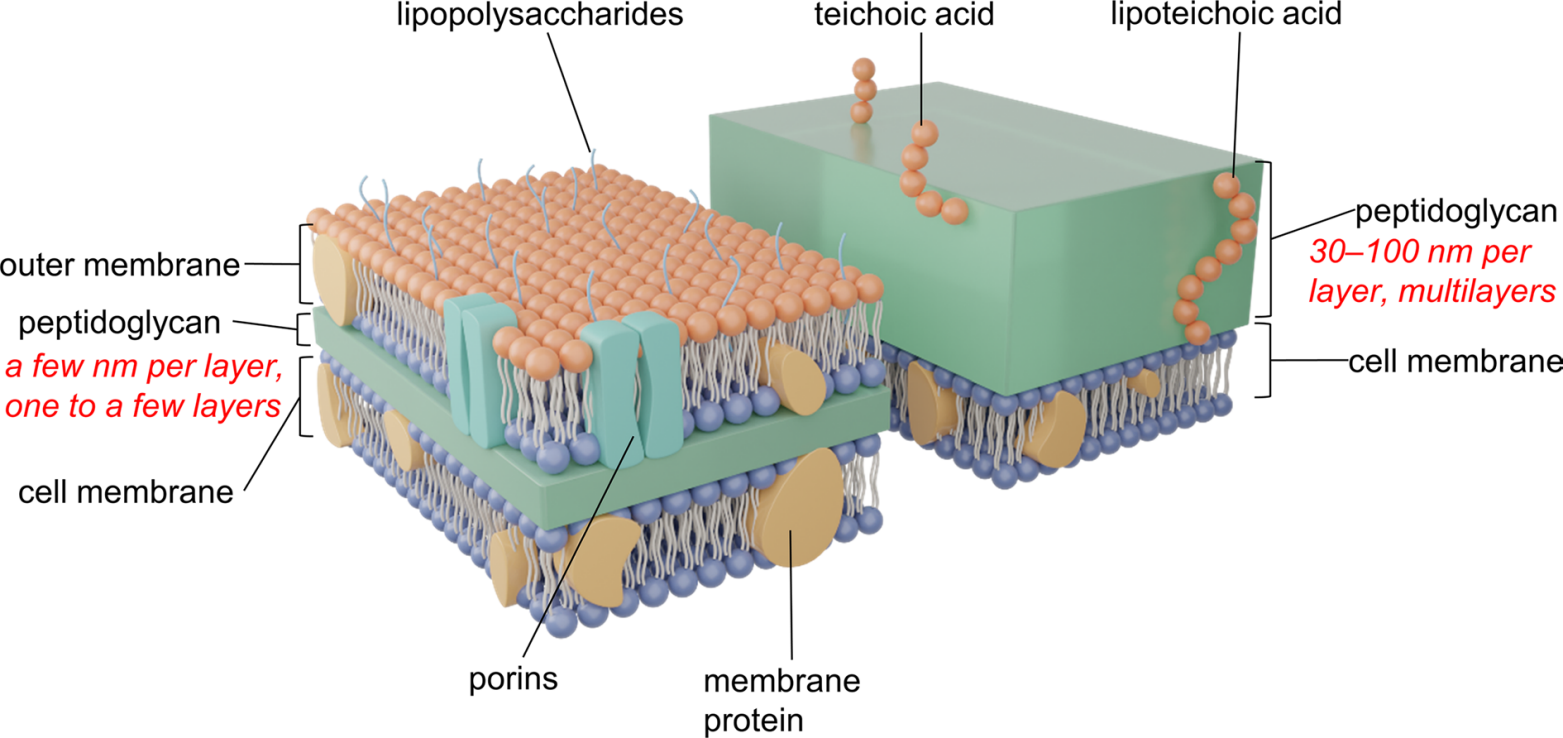
Cell wall structures of gram-negative (left) and gram-positive (right) bacteria

The structures of the cell walls of gram-positive and gram-negative bacteria are shown in Fig. 1. We aim to elucidate the key features of these membrane components in two well-studied model organisms: *Escherichia coli* (*E. coli*), a gram-negative bacterium and *Staphylococcus aureus* (*S. aureus*), a gram-positive bacterium. Gram-negative and gram-positive bacteria represent two distinct classes, characterized by fundamental differences in their cell envelope architecture. These disparities are primarily attributed to variations in lipopolysaccharides (LPS), phospholipid bilayers, and protein content. (A) *LPS*. One of the most striking differences between gram-negative and gram-positive bacteria lies in the composition and function of LPS, an integral component of the outer membrane of gram-negative bacteria. (B) *Phospholipid bilayers*. Both gram-negative and gram-positive bacteria possess a phospholipid bilayer, which forms the fundamental barrier between the cytoplasmic contents and the external milieu. However, their composition and arrangement differ significantly. Gram-negative bacteria exhibit a bilayer comprising mainly phospholipids, whereas gram-positive bacteria possess an additional thick layer of peptidoglycan. The presence of peptidoglycan in gram-positive bacteria provides structural support that makes it more difficult to puncture (thickness of 30–100 nm), while its absence in gram-negative bacteria contributes to a more flexible cell envelope (only a few nanometers-thick) [30]. (C) *Protein content*. The protein composition of bacterial membranes plays a crucial role in diverse cellular processes. Gram-negative bacteria typically exhibit a higher protein content within their cell membranes compared to gram-positive bacteria. This disparity arises from the abundance of outer membrane proteins, such as porins and efflux pumps, that facilitate the exchange of molecules with the external environment. In contrast, gram-positive bacteria tend to have a higher proportion of membrane-associated proteins involved in cell wall synthesis and maintenance. These differences emphasize distinct membrane functionalities, adaptability to environmental conditions, and interactions with host organisms. Understanding the unique characteristics of these membranes, as exemplified by *E. coli* and *S. aureus*, is essential for designing a universal antibacterial surface.

To address these challenges associated with the regular GLAD process for creating antibacterial surfaces, we propose a novel approach called Inverted GLAD (I-GLAD) [31]. This new technique aims to generate large-area, robust, and portable antibacterial surfaces.

## Methods

### I-GLAD fabrication process

**Fig. 2:**
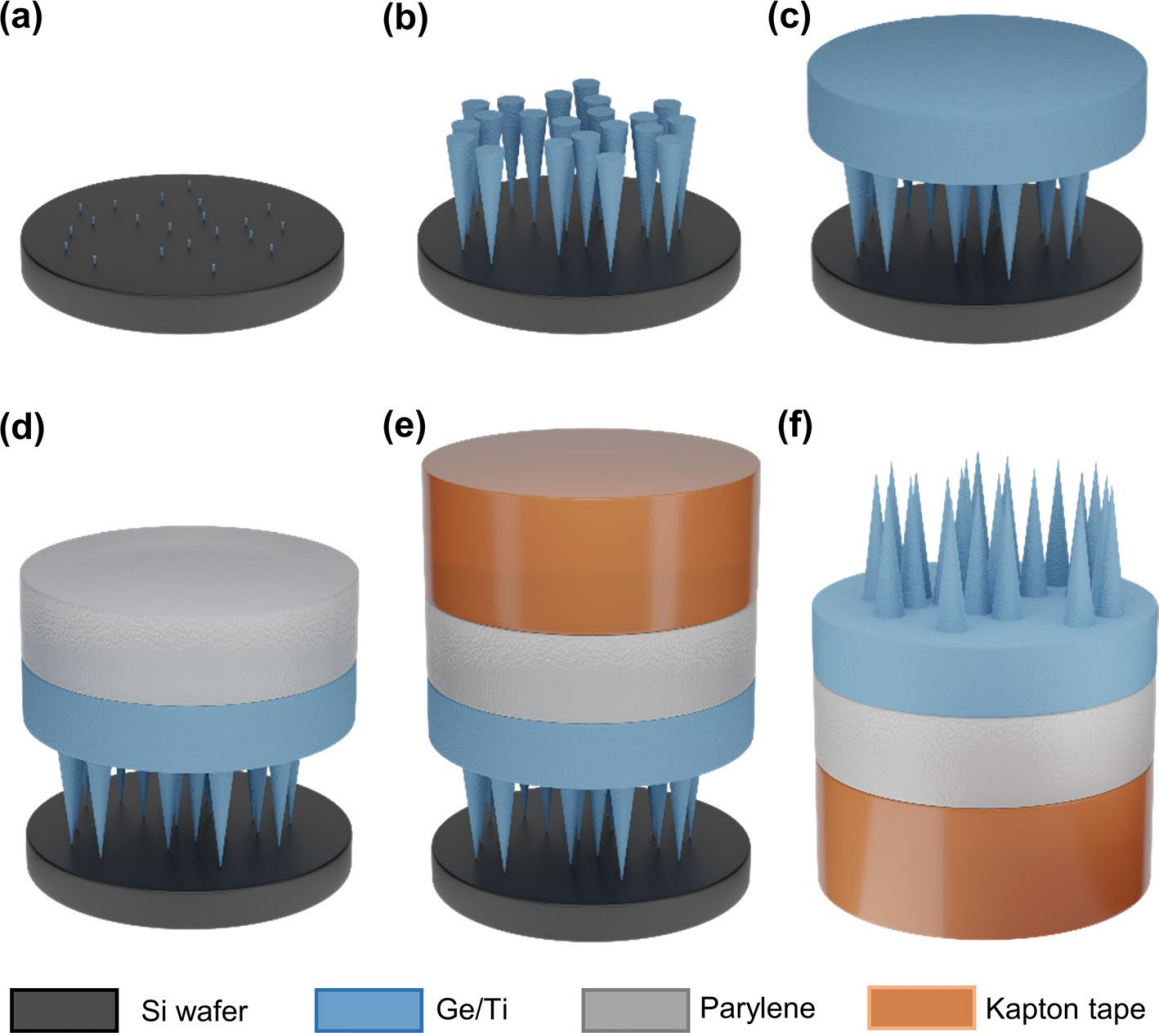
I-GLAD fabrication flow. **a** Natural seeds formed by *α*_*max*_. **b** Natural seeds grown out with *α*_*max*_. **c** Capping of ends of nanoneedles by lower *α*. **d** Application of a mechanical strengthening layer. **e** Application of a sticky superstrate. **f** Inverted nanoneedles

Figure 2 illustrates the fabrication flow for creating antibacterial surfaces with nanoneedles using I-GLAD. In a typical GLAD process, an e-beam evaporator operating under ultrahigh vacuum conditions (approximately 1.0e^−6^ Torr) with substrate tilt and rotation capabilities is utilized. We use Ge and Ti as the deposition materials in this process. A 4" silicon wafer serves as the substrate for growing the GLAD structures. The deposition process for both materials begins at an 85° incident angle (*α*_*max*_), resulting in the formation of natural seeds on the substrate, as depicted in Fig. 2a. The natural seeds cover approximately 30% of the substrate and are relatively spaced apart from each other [20]. The growth of the needle-like structures continues with the incident angle maintained at 85° for an additional nominal thickness of 1 µm deposition. As illustrated in Fig. 2b, the circumferences of the needles increase as the vapor deposition progresses, while the number of fully grown needles decreases due to shadowing. Once the lengths of the needles exceed 500 nm, the capping process commences. It involves reducing the incident angle to 65° for another nominal thickness of 0.5 µm deposition, followed by a final decrease to 0° for an additional nominal thickness of 0.5 µm, as shown in Fig. 2c.

After the GLAD process, the samples were further coated with a layer of parylene. This additional parylene layer enhances the mechanical strength of the thin film of nanoneedles. To achieve the desired thickness of ~ 20 µm for the parylene layer, approximately 10 mg of Parylene C monomers were used, as illustrated in Fig. 2d. The deposition was performed using an SCS vapor deposition system.

Finally, the entire structure is inverted by applying a 2-mil-thick Kapton tape over the films, as shown in Fig. 2e. The adhesion between the continuous top layer of the structure and the tape is stronger than the adhesion between the tips of the needles and the Si wafer. As a result, the surface with nanoneedle structures can be easily detached and inverted from the substrates.

### Characterization

**Fig. 3:**
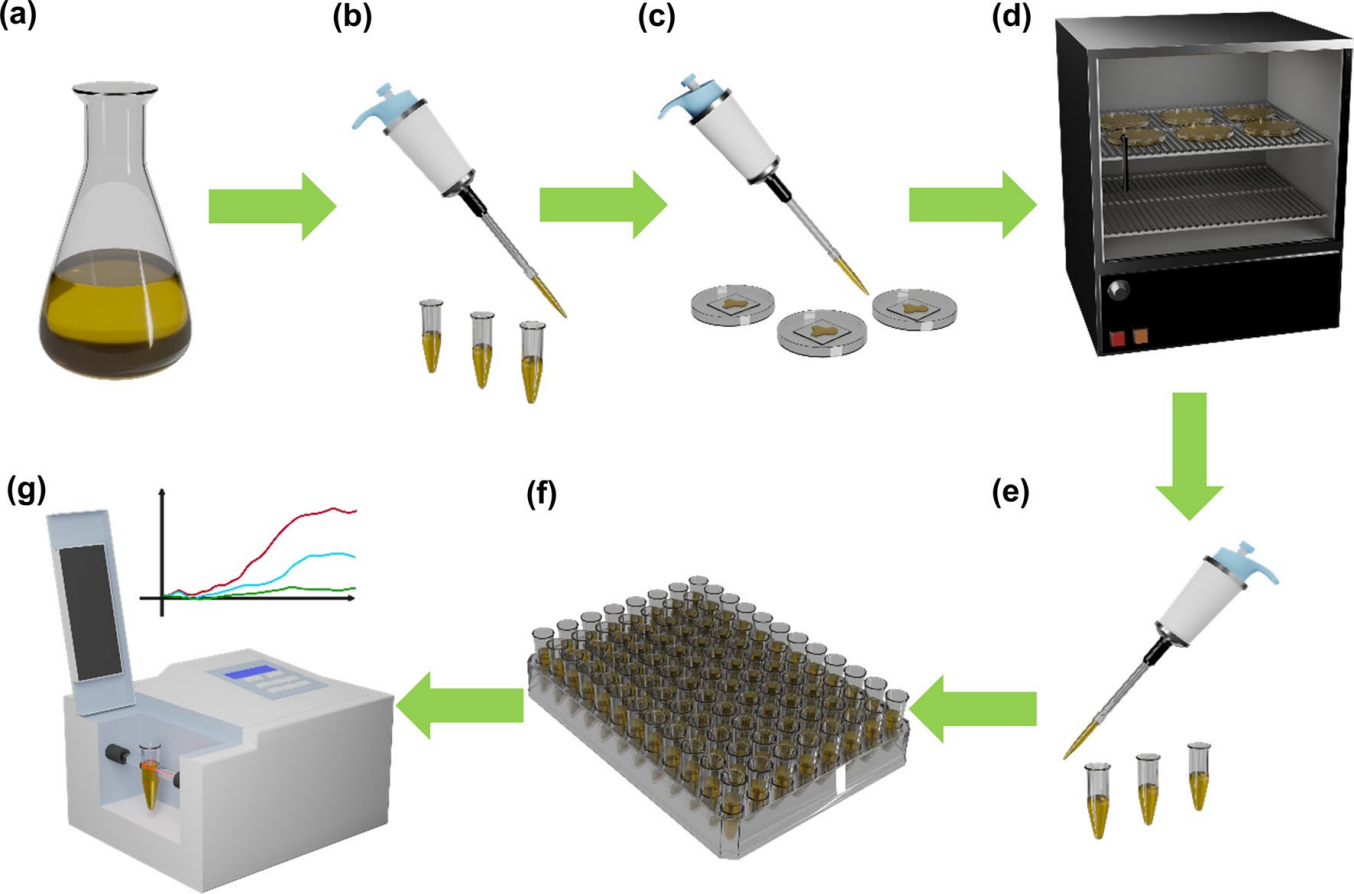
Bacteria test flow. **a** Cultivating bacteria. **b** Aliquots after dilution. **c** Applying bacteria on samples. **d** Incubation in the oven at 37° for 4 h. **e** Dilution after harvesting bacteria from samples. **f** Dispensing into test tubes. **g** Obtaining growth curves by spectrophotometer

Figure 3 illustrates the process of testing antibacterial properties. The experiment focuses on two representative bacteria, *Escherichia coli* (*E. coli*, a representative gram-negative bacterium) and *Staphylococcus aureus* (*S. aureus*, a representative gram-positive bacterium). *E. coli* strain BL21 (Thermo Fisher) was grown from glycerol stock (stored in − 80 °C) by inoculating Lysogeny Broth (LB) at 37 °C for 24 h until growth is saturated in LB. *S. aureus* was also grown in similar conditions. Following a protocol similar to previously published work [12], after 24 h, the *E. coli* and *S. aureus* in LB were diluted to an optical density (OD) of 0.1 [29, 32]. The bacteria were aliquoted onto the deposited samples at a volume of 10 µL using a standard micropipette (Rainin). Subsequently, these deposited samples harboring the bacteria were placed in a covered petri dish and incubated at 37 °C for 4 h in a static incubator. Following the incubation period, the bacteria were harvested using 10 µL of LB medium from the surfaces on which they had grown.

Each group of tests includes four samples: an I-GLAD sample, a continuous thin film (TF) of either Ge or Ti, a copper sheet, and a Kapton sheet. The I-GLAD samples were prepared using the fabrication process described earlier. The continuous thin films (500 nm) of Ge or Ti were prepared through conventional deposition within an e-beam evaporation system. Copper sheets are included as positive controls due to their known naturally occurring antimicrobial properties [5] (results not shown in the figures below). Kapton sheets, on the other hand, serve as negative controls, since they do not possess any antimicrobial properties.

For determining the growth curves, aliquots of the harvested bacteria were placed in a 96-well optical density plate (Greiner Bio-One) for spectroscopy. In parallel, 300 µL of LB were placed into each well in triplicate to each harvested *E. coli* and *S. aureus* samples. The 96-well plate also had LB blanks present for calibration. The spectrophotometer (SpectraMax M2, Molecular Devices) was programmed to maintain a temperature of 37 °C for 20 h, to shake the plate for 5 s before a OD reading took place, to read each well at 600 nm wavelength, and to read every 30 min. After 20 h, the raw data was analyzed by averaging the triplicate data, and the growth curves of the *E. coli* and *S. aureus* were obtained.

Scanning Electron Microscopy (SEM, Thermo Scientific Apreo) was used [33, 34] for imaging the I-GLAD samples, before and after the introduction of bacteria.

## Results and Discussion

### Theoretical analysis of I-GLAD parameters

**Fig. 4:**
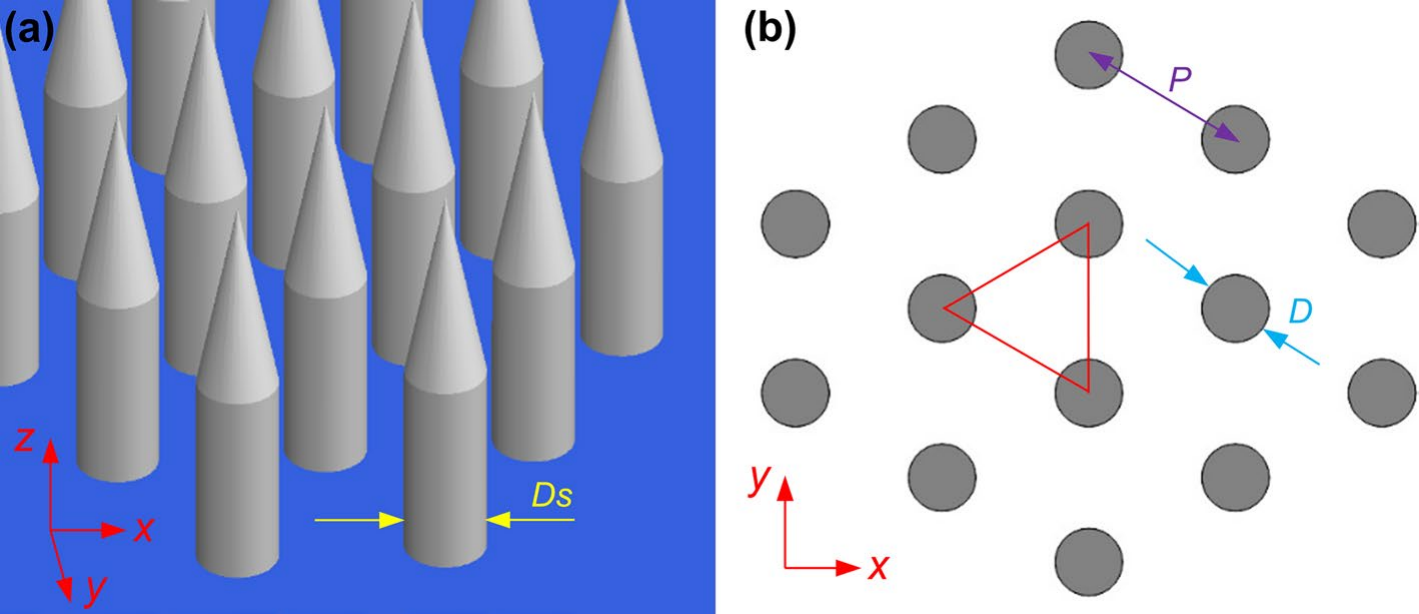
Equivalent model for I-GLAD nanoneedles. **a** 3D model. **b** 2D model (*x*–*y* plane)

Performing GLAD on an unpatterned, smooth and flat surface results in the formation of natural seeds, which turn into nano-structured needles as the deposition process continues. As the needles grow, their diameters increase due to the broadening effect. However, at a certain height, the diameter of the needles saturates. While the distribution of features in GLAD with natural seeds appears random, we approximate this surface with hexagonally packed nanoneedles (each comprised of a conical tip atop a cylindrical base) in our model, as depicted in Fig. 4a. The distribution of these cylinders in the *x*–*y* plane are shown in Fig. 4b, with the diameter of the feature (either the cone or the cylinder) denoted as *D* and the period between closest neighbors is *P*. The diameter of the cylinders is denoted as *D*_*s*_ (the subscript s for saturation). This depiction aims to encapsulate the essence of natural seed growth, wherein broadening occurs during the initial growth, and the diameter eventually saturates at a particular height [28].

Several studies have investigated the mechanisms behind the bactericidal effects of nanopatterned surfaces [10, 35]. A widely accepted mechanism involves the piercing and rupturing of bacteria cells. As these cells adhere to the nano-protrusions, the cell membrane stretches in the inter-protrusion regions. If the stretching reaches a critical point, the cells experience rupture. As the nano-protrusions are meticulously crafted to be sufficiently small for piercing bacteria, the sufficient volume of space between these nano-protrusions becomes pivotal in determining the rupture of bacterial cells. This space volume is defined by the ratio of protrusion feature size to period (*D*/*P*) and the height of the nano-protrusions (*H*). Considering that *D* and *P* are small enough to puncture bacteria, it's evident that a smaller *D*/*P* leads to a higher driving force or pressure on the cells. Conversely, when the *D*/*P* ratio becomes too high, indicating insufficient space volume among the nanostructures, the result is either no deformation or merely elastic deformation of bacteria cells. This outcome holds true regardless of the height of the nanostructures. When the *D*/*P* ratio falls below the critical point, it leads to creep deformation of the bacterial cells; depending on the height of the nano-protrusions, cell rupture occurs when their heights surpass 10% of the cell size [35]. With these design criteria, for ensuring the rupture of both gram-positive and gram-negative bacterial cells, the fabrication capabilities of I-GLAD are determined by the model.

The size-to-period ratio can be estimated from the model. Utilizing the empirical equation, the percentage coverage of GLAD films, solely contingent on the incident angle *α*_*max*_, can be estimated [20]. Applying a purely geometrical approach, the percentage coverage can also be calculated as (taking the triangle as the unit cell). By combining these two equations, we can estimate the ratio of *D*_*s*_/*P* for saturated needles:

1 $${D}_{s}/P=\sqrt{6\sqrt{3}\left.cos({\alpha }_{max}\right)/\pi (1+cos\left({\alpha }_{max}\right))}$$

**Fig. 5:**
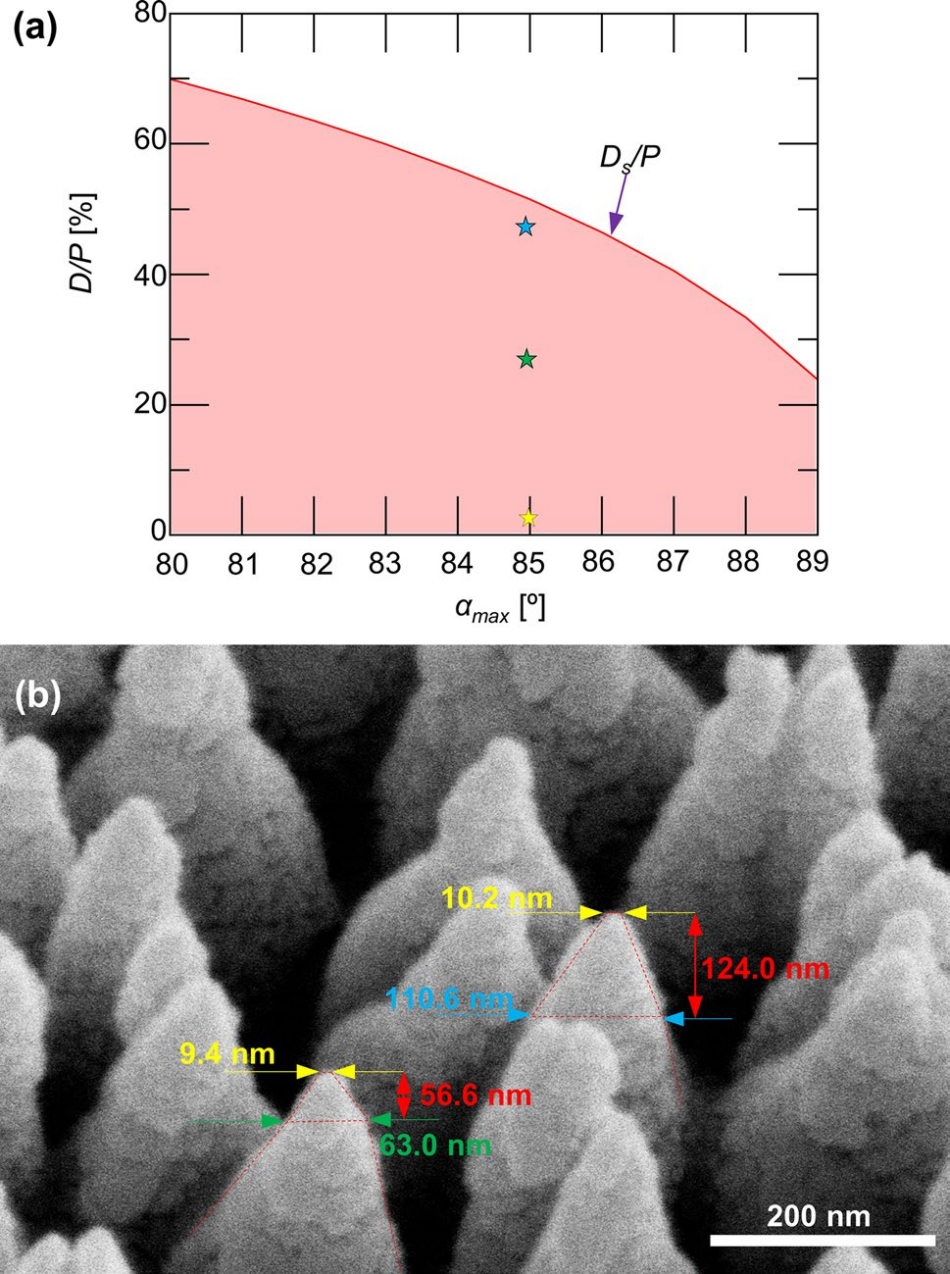
**a** *D*/*P* of I-GLAD nanoneedles as a function of the to the maximum incident angle *α*_*max*_. **b** SEM image (45° tilted) of zoomed-in nanoneedles for measurements

Due to the conical-cylindrical shape of the needles, the value of *D* is not uniform along the *z*-direction. The size of the seeds (the tips of the needles) can be as diminutive as sub-10 nm. Consequently, the *D*/*P* ratio ranges between nearly 0 to *D*_*s*_/*P*. In Fig. 5a, the relationship between the range of *D*/*P* ratio (shadow region) is displayed concerning *α*_*max*_, with a maximum incident angle exceeding 80º. With I-GLAD, nanoneedles can easily attain the optimal *D*/*P* ratio for inducing bacteria ruptures. I-GLAD maintains the ability to achieve desired nanoneedle heights *H*, since the heights of the needles when the diameter saturates is over 1 µm at high incident angles [31].

Figure 5b shows a detailed examination of the I-GLAD nanoneedles with key parameters in the zoomed-in image. The 45-degree-tilted SEM image reveals conical needle shapes with varying diameters along the needles, which makes the direct measurement of diameters impossible from top-view images. Top-view images are instrumental in determining average periods (*P*, averaged 227.69 nm), while 45-degree-tilted images aid in measuring diameters (*D*) since the dimensions in the *x*-direction remain undistorted. Heights (*H*) can also be measured from the 45-degree-tilted SEM image and adjusted by multiplying the tilting factor. The measured data points, the stars as shown in Fig. 5a, illustrate *D*/*P* ratios ranging from 4 to 48% from the needle tip to *H* = 124 nm, all well within the theoretical range. The color of the measurements in Fig. 5b matches the color of the stars in (a). Notably, the heights of the needles, exceeding 100 nm and maintaining sub-100 nm dimensions, are crucial for effectively puncturing gram-positive bacteria.

### I-GLAD samples

**Fig. 6:**
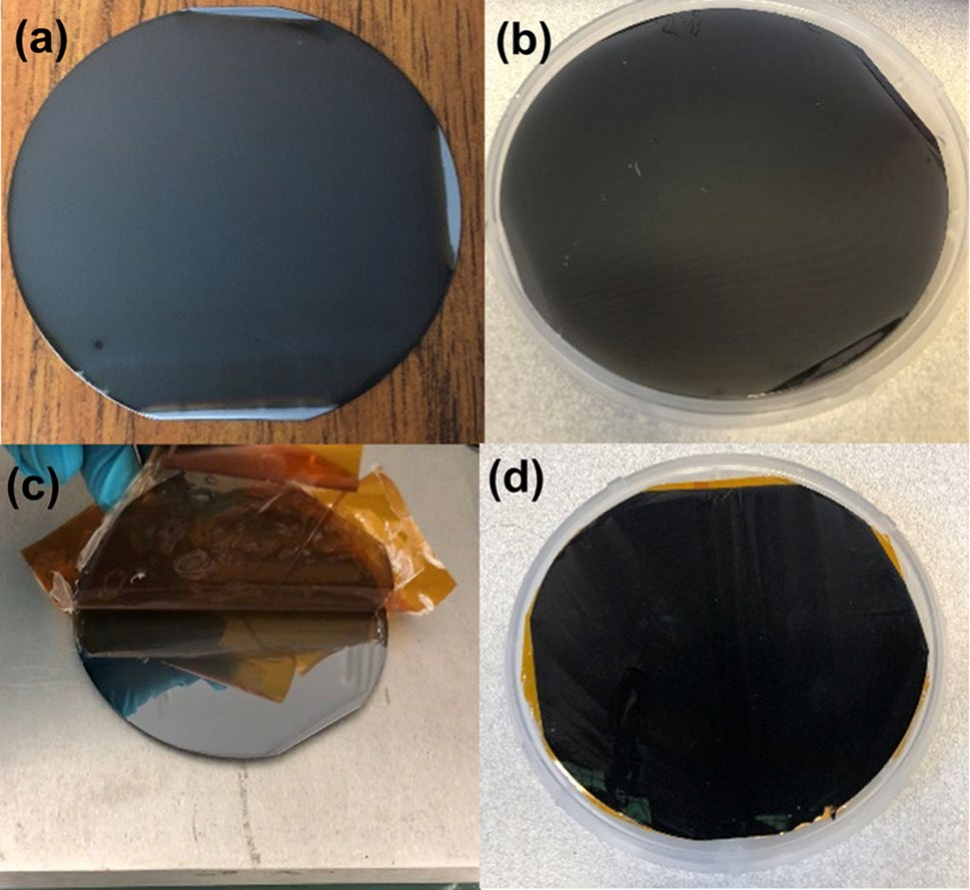
Pictures of I-GLAD samples. **a** After GLAD. **b** After adding the mechanical strengthening layer. **c** Inverting process on a hot plate (temperature 100 °C) (d) I-GLAD sample with nanoneedles facing up

Figure 6 shows pictures of actual I-GLAD samples captured at various stages during the fabrication. An interference pattern is discernible after the application of the transparent parylene film as the mechanical strengthening layer to the sample, as shown in Fig. 6b. Figure 6c depicts the inverting process occurring on a hotplate set at 100 ºC. The needle layer is autonomously released due to the difference in the coefficient of thermal expansion between the Si wafer and the Ge/Ti needles (each featuring sub-10 nm contact diameters). No external force is necessary for this inverting process. The resulting inverted sample, with needle tips facing upward on the wafer-size sample, appears black, as shown in Fig. 6d.

**Fig. 7:**
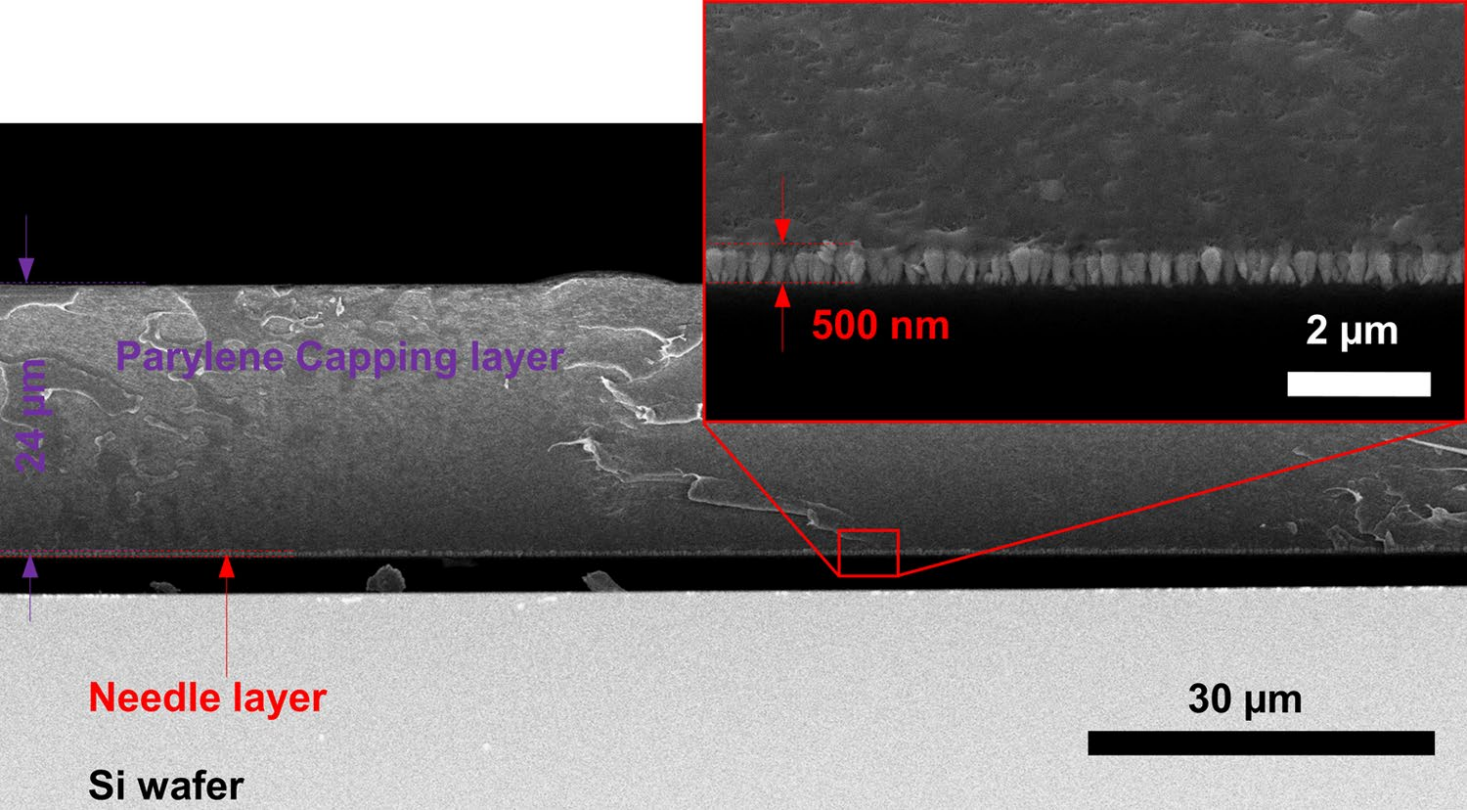
SEM images of Ge I-GLAD nanoneedles before inverting

Figure 7 shows scanning electron micrographs of the cross section of an I-GLAD sample before inversion. The thicker layer of parylene is around 48 times the of the height of the needle layer, which provides the mechanical support to keep the needles at the same plane. Due to the two capping layers (Ge/Ti and parylene), the wafer-sized area needle arrays are crack-free and ready for transportation. Alternative materials, such as polyimide, are available for this capping process.

**Fig. 8:**
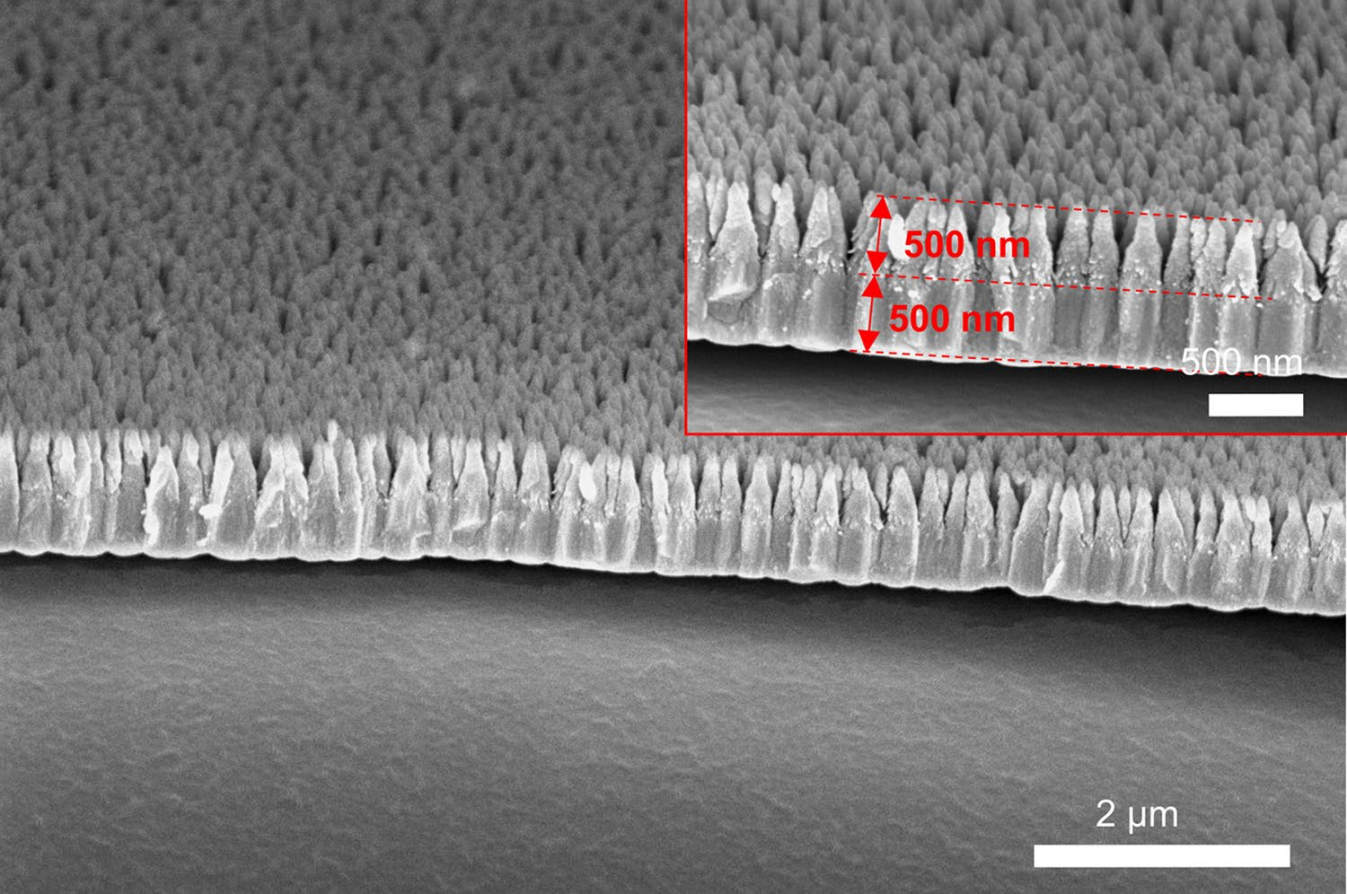
SEM images of Ge I-GLAD Nanoneedles after inverting

Figure 8 shows the zoomed-in Ge I-GLAD nanoneedles. With the recipe mentioned, the needles are very well separated at the tips, grew out and eventually merged to form a continuous film. The lengths of the needles are around 500 nm, which are controllable depending on the deposition rate and time. The spaces among the needles granted the space for the bacteria on top of the surfaces to dangle and be disintegrated. The thickness of the capping layer using normal deposition is also around 500 nm.

### Antibacterial test results

**Fig. 9:**
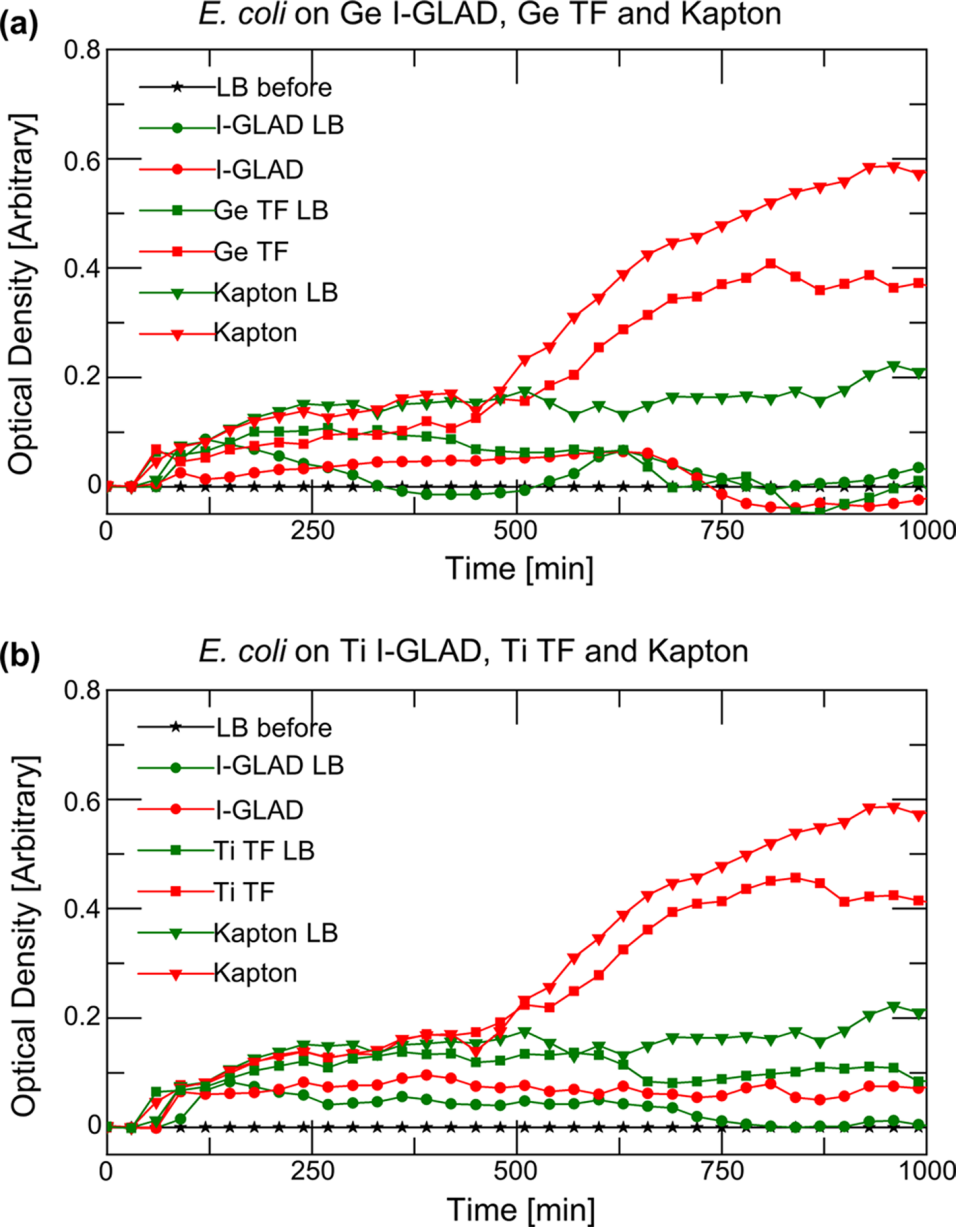
*E. coli* growth curves on I-GLAD, thin film (TF) samples of **a** Ge. **b** Ti and Kapton tape control

Figure 9 shows the growth curves of *E. coli* on different samples. The LB media for cultivating the bacteria is demonstrated to be sterile before use (black line with star symbols). All the sample surfaces are also tested using pure LB for sterilization before the application of the bacteria, and the green curves in the figures are reflecting that the surfaces are bacteria-free. The growth patterns of *E. coli* are differentiated on the samples, as the red curves indicate in the figures. Antibacterial performance of the I-GLAD samples for both Ge and Ti (with red circle symbol) are observed in the figures, with low optical densities throughout the measuring periods. For Ge thin film and Ti thin film (with red square symbol), there is *E. coli* growth shown in the curves. This shows that both Ge and Ti are not naturally antibacterial; the antibacterial properties by the corresponding I-GLAD Ge and Ti samples are due to the nanostructures of the nanoneedles. Similarly, the positive control of Kapton sheets (red down triangle symbol) shows the growth of *E. coli* occurred on them as well.

**Fig. 10:**
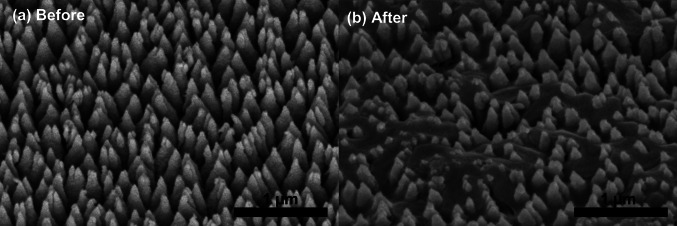
SEM images of I-GLAD nanoneedles before and after the introduction of *E. coli*

Figure 10 shows the I-GLAD sample before and after the application of *E. coli*. The samples were tilted 45° while imaging. It is clear the tips of the needles are sub-10 nm and very well separated from each other. The bacteria were fully punctured with the tips of the needles through the bacteria, which reflects the killing mechanism of the I-GLAD nanoneedles.

**Fig. 11:**
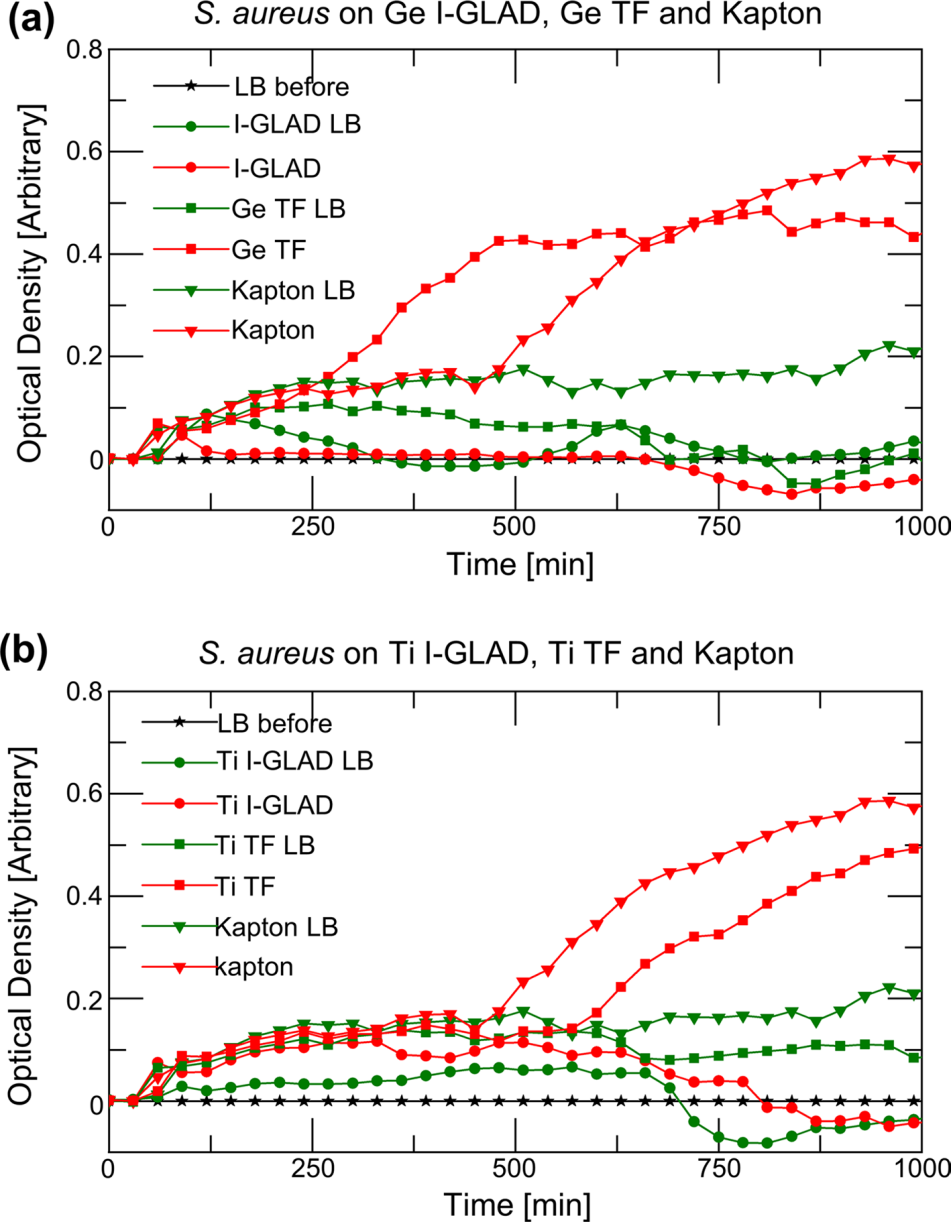
*S. aureus* growth curves on I-GLAD, thin film (TF) samples of **a** Ge. **b** Ti and Kapton tape (control)

Figure 11 shows the growth curves for *S. aureus* on I-GLAD samples. The results are showing the same trends of the sample against *S. aureus* as they are against *E. coli*. I-GLAD samples are showing no growth of *S. aureus* on top of neither Ge nor Ti sample, while the growth of *S. aureus* on the corresponding continuous films are observed.

**Fig. 12:**
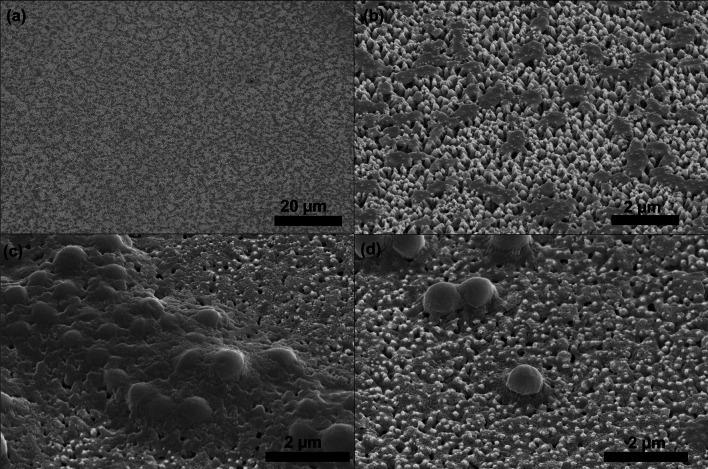
SEM images of I-GLAD nanoneedles after application of *S. aureus*. **a** A large area of needles with bacteria deposition. **b** *S. aureus* decomposed. **c** and **d** coccoid *S. aureus* being punctured with cytoplasm

Figure 12 shows some coccoid *S. aureus* on I-GLAD surface. There is a biofilm layer at the base of the bacteria; the biofilm could be cytoplasm showing the bacteria were punctured (there is salt crystallization in this area, so the surfaces of the needles look rough and the spacing between needles are partially filled in). Figure 12b shows the decomposed *S. aureus*.

While antibacterial surfaces such as cicada wings are known to primarily kill gram-negative bacteria, I-GLAD surfaces are showing antibacterial behavior for both gram-positive and gram-negative bacteria, regardless of the materials of the nanoneedles. This broad-spectrum effectiveness can be attributed to the size of the tips of the I-GLAD nanoneedles, which have diameters of less than 10 nm, while cicada wings feature nanocolumns with diameters of approximately 90 nm. Bacteria residing on nano-protrusions with smaller *D*s experience elevated pressure, leading to the deformation and rupture of their cell walls, even for gram-positive bacteria with thicker cell walls. This mechanism for physically eliminating bacteria is an active area of research.

## Conclusions

This paper presents I-GLAD, a straightforward, scalable, and cost-effective method for creating antibacterial surfaces. By leveraging natural seeds in GLAD, I-GLAD streamlines the fabrication process and allows one to produce nanoneedles with wide range of *D*/*P* ratios. The nanoneedles produced by I-GLAD feature finer tips of sub-10 nm, which exhibit physical bactericidal properties not only against gram-negative bacteria, but also gram-positive bacteria with thick cell walls. The experimental data and SEM images show extremely encouraging results for physically neutralizing both gram-negative (*E. coli*) and gram-positive (*S. aureus*) bacteria. Furthermore, I-GLAD antibacterial surfaces have the potential to be applied to various hospital surfaces (for example, catheters and ventilators) and daily surfaces.

## Data Availability

The datasets used and/or analyzed during the current study are available from the corresponding author on reasonable request.

## Abbreviations

I-GLAD: Inverted glancing angle deposition
GLAD: Glancing angle deposition
*E. coli*: *Escherichia coli*
*S. aureus*: *Staphylococcus aureus*
3D: Three-dimensional
PVD: Physical vapor deposition
LB: Lysogeny broth
SEM: Scanning electron microscopy

## Code availability

Nor applicable.
